# Modeling and Manufacturing of a Micromachined Magnetic Sensor Using the CMOS Process without Any Post-Process

**DOI:** 10.3390/s140406722

**Published:** 2014-04-11

**Authors:** Jian-Zhi Tseng, Chyan-Chyi Wu, Ching-Liang Dai

**Affiliations:** 1 Department of Mechanical Engineering, National Chung Hsing University, Taichung, 402 Taiwan; E-Mail: a16824795@hotmail.com; 2 Department of Mechanical and Electro-Mechanical Engineering, Tamkang University, Tamsui, 251 Taiwan; E-Mail: ccwu@mail.tku.edu.tw

**Keywords:** magnetic sensor, magneto-transistor, CMOS

## Abstract

The modeling and fabrication of a magnetic microsensor based on a magneto-transistor were presented. The magnetic sensor is fabricated by the commercial 0.18 μm complementary metal oxide semiconductor (CMOS) process without any post-process. The finite element method (FEM) software Sentaurus TCAD is utilized to analyze the electrical properties and carriers motion path of the magneto-transistor. A readout circuit is used to amplify the voltage difference of the bases into the output voltage. Experiments show that the sensitivity of the magnetic sensor is 354 mV/T at the supply current of 4 mA.

## 1. Introduction

The operation of precision equipment is always affected by the magnetic field, which deflects the transmission path of electrons or holes, and changes the resistance of magneto-resistive materials. These effects caused by the magnetic field can affect the performance of an instrument or make it export unreliable data. Therefore, correcting the magnetic field signal is very important. To resolve this problem, this study designs a micro-magnetic sensor to detect the magnetic field variation in an environment. The magnetic microsensor can also be applied to protecting human health as well as in monitoring the magnetic field of equipment environments. The magnetic field affects human health by deflecting the transmission path of charged particles in human blood. This can make people feel dizzy or develop a metallic taste in their mouths [1].

The magnetic microsensor in this study is implemented using micro-electromechanical system (MEMS) technology. That has the advantage of small size, low cost and ease of mass production. Moreover, MEMS chips can be merged in portable devices such as cell phones and laptops. Some magnetic microsensors have been developed by MEMS technology [2]. Nazarinejad *et al.* [3] developed a giant magneto-impedance (GMI) magnetic sensor as a magnetic switch. The GMI sensor had both meandering and straight film structure, and the main material was Co_73_Si_13_B_15_ with a maximum impedance at 10 Gauss. Wattanasarn *et al.* [4] designed a 3-dimensional Lorentz sensor using MEMS technology. It was composed of three thin piezoresistive cantilevers. One of the z-axis parts was bent by Cu layer deposition and a thermal annealing process. The resolution of the sensor was 1 mT with a power consumption of less than 11 mW. Estrada [5] fabricated a 3-dimensional Hall sensor using MEMS technology and a silicon on insulator (SOI) wafer. The Hall sensor was implemented during repeated etching and deposition of metal and polyimide layers. After combination of three identical components, the sensitivity of each element was 0.02 mV/T. Ristic *et al.* [6] designed a lateral magneto-transistor using CMOS technology. It was an NPN (p-doped semiconductor between two n-doped layers) transistor using an n-well as the body. The principle in this magneto-transistor is the imbalance between the currents of both collectors deflected by the magnetic field. Furthermore, Leepattarapongpan *et al.* [7] also designed a lateral magneto-transistor using CMOS technology. This was a PNP (n-doped semiconductor between two p-doped layers.) transistor based on an n-type substrate. The magneto-transistor was connected with a resistor in series, then the node voltage between the base and collector electrodes was varied, because of the current deflected by the magnetic field. Under a 5 mA supply current, the relative sensitivity of this magneto-transistor was 110 mV/T. The magneto-transistor could detect the magnetic field at the *y* and *z*-axes at the same time. Caruntu *et al.* [8] investigated the relationship between the ratio of electrode width (W) to spacing of electrodes (L) and relative sensitivity. This research strengthens the relative sensitivity of the magneto-transistor by changing the material of the substrate. The Hall mobility based on a GaAs substrate was much larger than that based on a silicon substrate, and the relative sensitivity of the magneto-transistor was dramatically strengthened by using the GaAs substrate. The relative sensitivity of the magneto-sensitivity was 5.5%/T. There are many different types of magnetic sensors [3–8]. These sensors were fabricated using either particular materials or difficult processes, and they required a high voltage supply or a non-traditional silicon substrates.

Various microdevices have been fabricated using the commercial CMOS process [9–13]. Micro-devices manufactured by this process have the potential for integration with circuitry on-a-chip [14–16]. In this work, we develop a magnetic microsensor using the commercial CMOS process. Microsensors and microactuators fabricated by the CMOS process usually require a post-process [17–20] to add functional films or to release suspended structures [21–24]. Some magnetic sensors were designed in CMOS technology [25–27], and they all needed a post-process. In this work, the fabrication of the magnetic sensor is fully compatible with the commercial CMOS process without any post-process. The advantages of the magnetic sensor include easy fabrication and low cost. The magnetic microsensor is designed based on a magneto-transistor, which is a PNP bipolar structure which can be utilized in portable electronic devices or cell phones because of the low supply voltage and the reduced power dissipation resulting from the decrease in current supply. The output signal of the magnetic sensor is amplified by a readout circuit. This work strengthens the sensitivity and sensing range of the magneto-transistor by adjusting the topology and electrodes.

## 2. Design and Simulation of the Magnetic Microsensor

**Figure 1.**
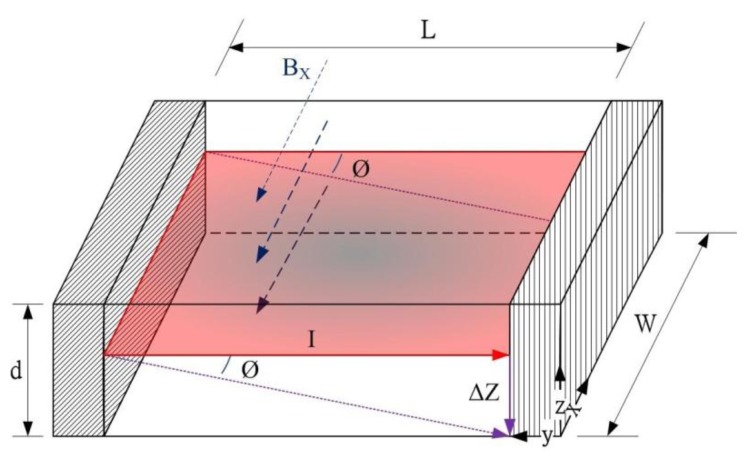
Current deflected by the magnetic field of the x axis.

The magnetic sensor is designed based on a magneto-transistor. The magneto-transistor depends on the Hall effect, which is more evident in semiconductors than in conductors. The proposed magneto-transistor adopts silicon as a substrate. Figure 1 illustrates the current *I* deflected to the −*z* direction (*ΔZ*) by the magnetic field of the *x* axis, and the Hall angle is *Ø*. When the displacement of current is *L* and the major carrier is holes, the relationship between displacement and the angle of deflection is given by:

(1) $$\Delta Z=L\cdot \tan Ø=\mu_{P}\cdot B_{X}\cdot L$$

where *L* is the displacement of current, *B_X_* is the magnetic field of the *x* direction, *Ø* is the Hall angle deflected by *B_X_*, and *μ_P_* is the Hall mobility.

The current *I* is transmitted from the left electrode to the right electrode, as shown in Figure 1. When the magnetic field is applied in the *x* axis, the current *I* is deflected to the −*z* direction and part of current (*ΔI*) is not received by the right electrode, as shown in Figure 1. The loss current of *ΔI* can be expressed as [8]:

(2) $$\Delta I=\frac{L}{d}\cdot \mu_{P}\cdot B_{X}\cdot L\cdot G\cdot I$$

where *G* is the geometrical correction factor, *d* is the depth of electrode. The right electrode is connected in series with a resistor *R*, and the voltage difference of *V_b-b_* caused by the magnetic field is given by [8]:

(3) $$V_{b-b}=\frac{L}{d}\cdot \mu_{P}\cdot B_{X}\cdot L\cdot R\cdot G\cdot I$$

where *R* is the resistance connected with the electrode. According to Equation (3), the voltage difference of *V_b-b_* generates a change as the magneto-transistor senses the magnetic field of *B_X_*.

**Figure 2.**
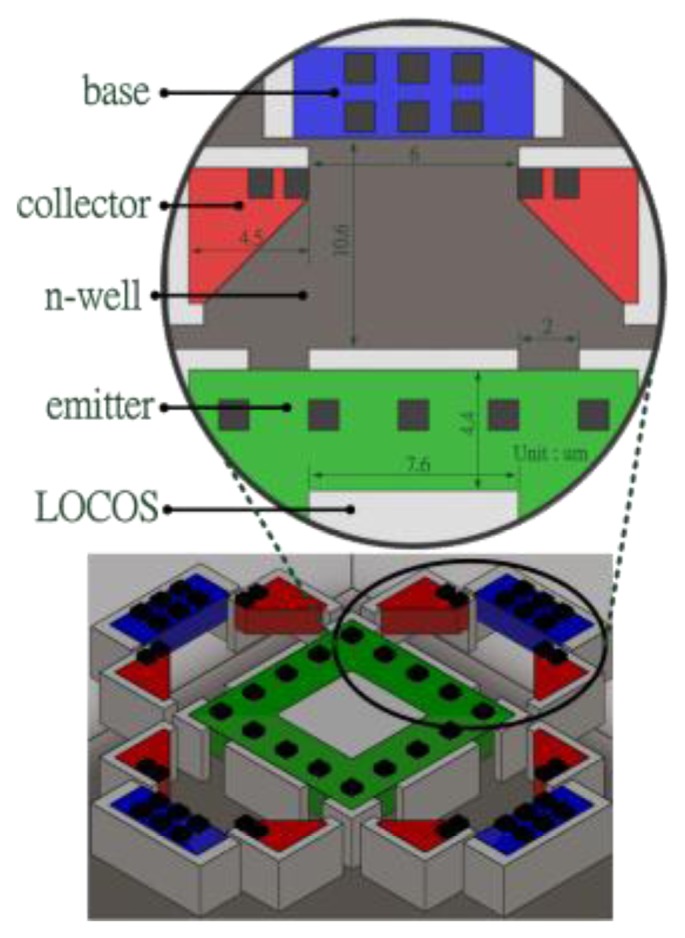
Isometric view of the magneto-transistor.

The magneto-transistor in this study uses PNP structure, as shown in Figure 2. The proposed magneto-transistor includes one p-type emitter, eight p-type collectors and four n-type base electrodes. The design uses local oxidation of silicon (LOCOS) of an undefined area around the emitter electrode bevel edge [28] to resist transmission of current, and also uses LOCOS inside the emitter center to reduce the vertical pn-junction effect. Each base electrode is connected with a resistance in series, so that we can measure both base electrodes opposite each other to readout the magnetic signal of the x and y axes.

**Figure 3.**
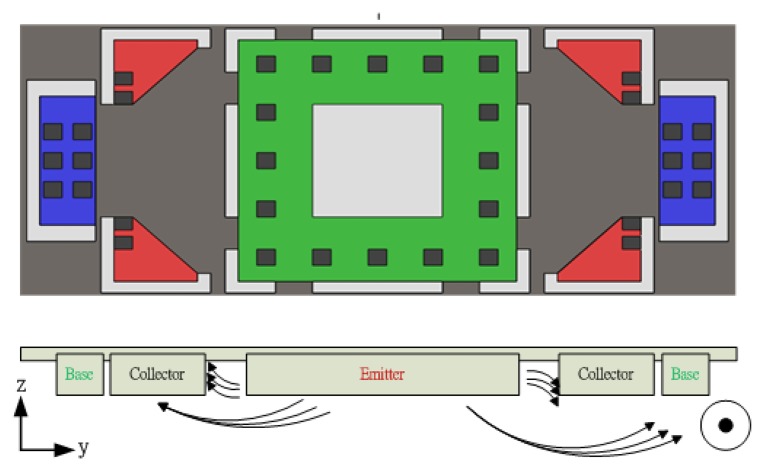
Current of magneto-transistor deflected by x-axis magnetic field.

Figure 3 presents the design of the magnetic microsensor for detecting the magnetic field in the x axis. The current transmitting from the emitter to the collector is deflected down and across the collector electrode to the base electrode. Thus the current of the right base electrode is increased and the current of the left one is decreased. Therefore, we can distinguish positive or opposite magnetic fields in the x axis through the imbalance of both electrodes.

**Figure 4.**
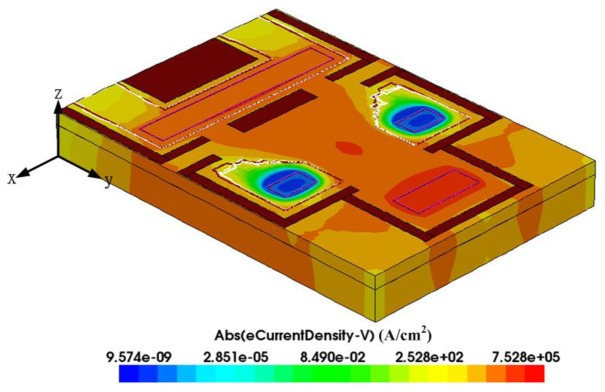
Carriers density of the magneto-transistor in a 250 mT magnetic field.

The FEM software Sentaurus TCAD is employed to simulate the motion of carriers in the magneto-transistor. To reduce the simulation time and model size, only one-quarter of the magneto-transistor is established. The Delaunay triangulation method is used to mesh the model, and there are about 3.7 million elements. We use the Poisson electron hole method to couple electoral field, magnetic field and other effects. The Bank/Rose nonlinear solver is adopted to converge the simulation model. Figure 4 shows the distribution of current density for the magneto-transistor with a magnetic field of 250 mT. In this simulation, the bias voltage of the emitter is 2.6 V, and the current of the emitter is 4 mA. The results showed that the current of the bases increased 390 nA under the magnetic field of 250 mT.

**Figure 5.**
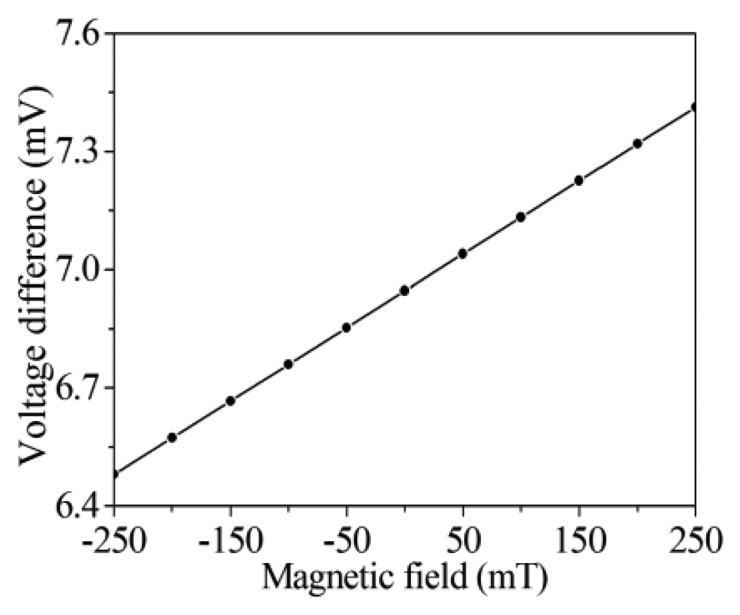
Simulation results of voltage difference of the bases for the magneto-transistor.

To characterize the voltage difference of the bases, the magneto-transistor is simulated with different magnetic fields in the *x* axis. Figure 5 shows the simulation results of the voltage difference of the bases for the magneto-transistor. In this investigation, the voltage and current of the emitter are also 2.6 V and 4 mA, respectively. The simulation results showed that the voltage difference of the bases increased from 6.5 to 7.4 mV when the magnetic field changed from −250 to 250 mT.

**Figure 6.**
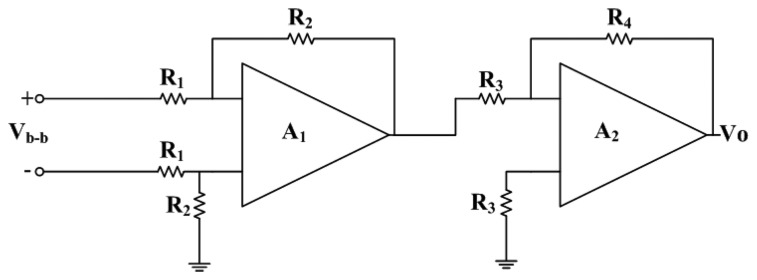
Readout circuit for the magnetic microsensor.

**Figure 7.**
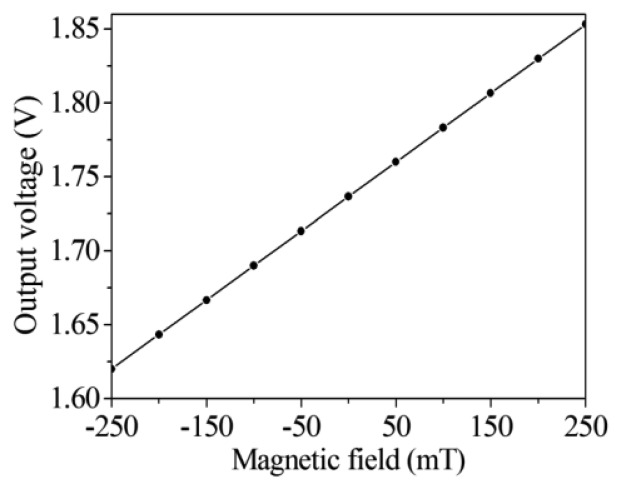
Output voltage of the readout circuit.

As shown in Figure 5, the voltage difference of bases in the magneto-transistor is small. To enlarge the voltage difference of bases, a readout circuit is employed. Figure 6 illustrates the readout circuit of the magnetic sensor, where *V_b-b_* is the voltage difference of the bases of the magneto-transistor and *V_0_* is the output voltage. The circuit is composed of two amplifiers and six resistors. The first stage amplifier of *A_1_* is used to enlarge the voltage difference of the bases *V_b-b_*. Then, the second stage amplifier of *A_2_* is utilized to strengthen the output signal of the amplifier *A_1_* again. The output voltage of the readout circuit is given by [29]:

(4) $$V_{0}=\left(\frac{R_{2}\cdot R_{4}}{R_{1}\cdot R_{3}}\right)V_{b-b}$$

where *R_1_*, *R_2_*, *R_3_* and *R_4_* are the resistors of the readout circuit, and *V_0_* is the output voltage of the readout circuit. According to Equation (4), we know that the gain of the readout circuit depends on the resistors. In this design, the resistors *R_1_*=1 kΩ, *R_2_*=50 kΩ, *R_3_* =10 kΩ and *R_4_* =50 kΩ are adopted. Substituting the values of the resistors and the voltage difference of the bases in Figure 5 into Equation (4), the output voltage of the readout circuit can be obtained. Figure 7 shows the evaluated results of the output voltage of the readout circuit for the magnetic sensor. The results showed that the output voltage of the magnetic sensor changed from 1.62 to 1.85 V as the magnetic field varied from −250 to 250 mT.

## 3. Fabrication of the Magnetic Microsensor

**Figure 8.**
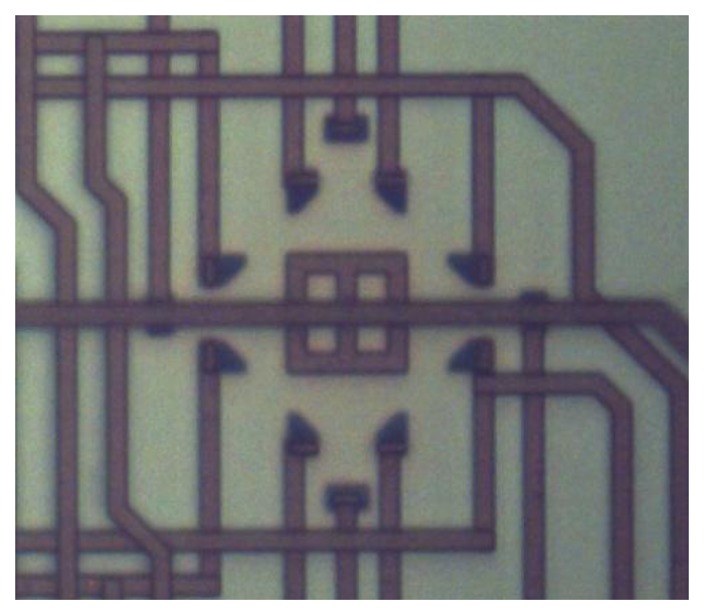
Optical image of the magnetic sensor after the CMOS process.

**Figure 9.**
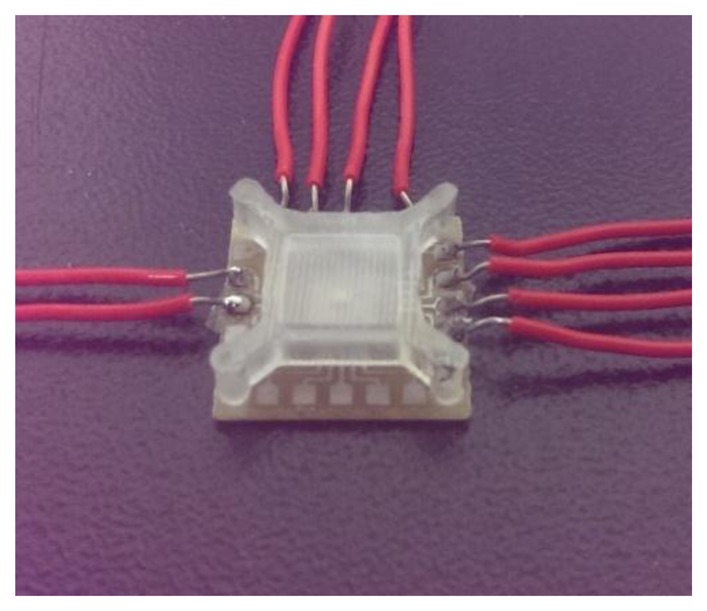
Optical image of the magnetic sensor after completion of packaging.

This study implemented the design and fabrication of the proposed magnetic senor using the commercial 0.18 μm CMOS process of the Taiwan Semiconductor Manufacturing Company (TSMC, Taipei, Taiwan). The first step was light doping the phosphorus into a p-type silicon substrate as an n-well by ion plasma implantation. After the body (n-well) of the magneto-transistor was defined, the emitter and collector parts were light doped with boron; the bases were light doped with arsenic. Then the emitter and collector were heavily doped again by boron implantation; the bases were heavily doped in the same way as the n-well. When the above process was finished, the magnetic sensor was practically completed. Figure 8 shows an optical image of the magnetic sensor after completion of the CMOS process. Finally, the chip of the magnetic sensor was wire-bonded on a printed circuit board by an aluminum wire bonder. In order to prevent impact, dust and other outside interference, it was packaged in an acrylic protective cover. Figure 9 shows an optical image of the magnetic sensor after completion of wire-bonding and packaging.

## 4. Results

**Figure 10.**
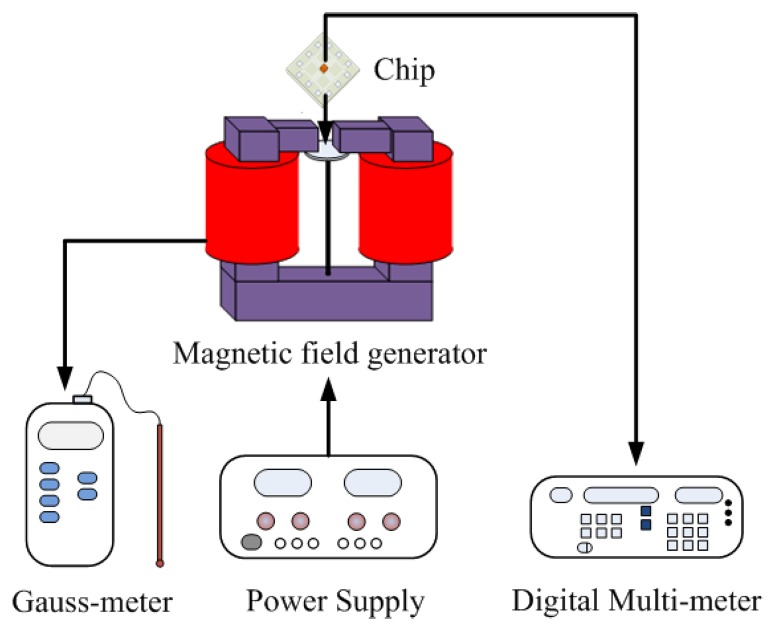
Measurement set-up for the magnetic sensor.

A magnetic field generator, a Gauss-meter, a digital multi-meter, and power supplies were used to test the performances of the magnetic sensor. Figure 10 illustrates the measurement set-up for the magnetic sensor. The magnetic field generator was used to supply a magnetic field to the magnetic sensor chip. The Gauss-meter was employed to calibrate the value of magnetic field generated by the magnetic field generator. The digital multi-meter was utilized to record the output signal of the magnetic sensor. The power supplies provided power to the magnetic field generator and magnetic sensor.

**Figure 11.**
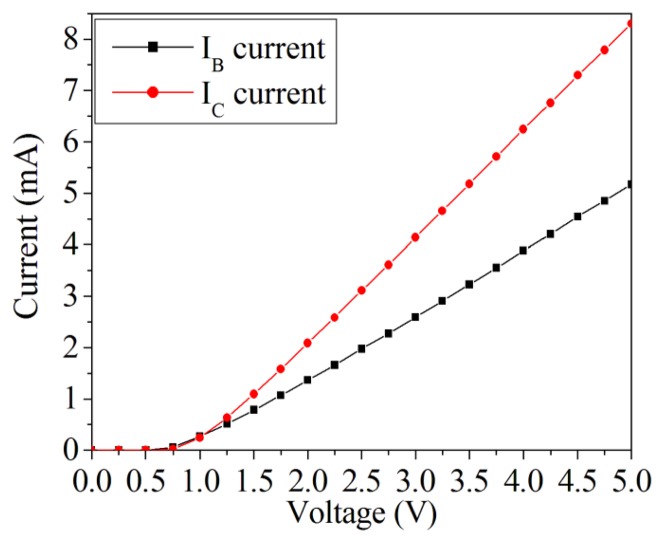
I-V characteristic of the magneto-transistor.

Before detecting magnetic field, the characteristics of the magneto-transistor in the sensor chip were tested without magnetic field. Figure 11 shows the characteristic relation between the current and voltage of the magneto-transistor, where *I_B_* is the current of base and *I_C_* is the current of collector. In this measurement, the collector and base electrodes were similarly connected with 1 kΩ resistance in series. The results revealed that the magneto-transistor started operation at 0.75 V, resulting from the driving voltage of silicon pn-junction 0.7 V. The current of collector increased from 0 to 8 mA as the supply voltage changed from 0.75 to 5 V. The current of base varied from 0 to 5 mA as the supply voltage increased from 0.75 to 5 V. The current of the collector exceeds that of the base.

**Figure 12.**
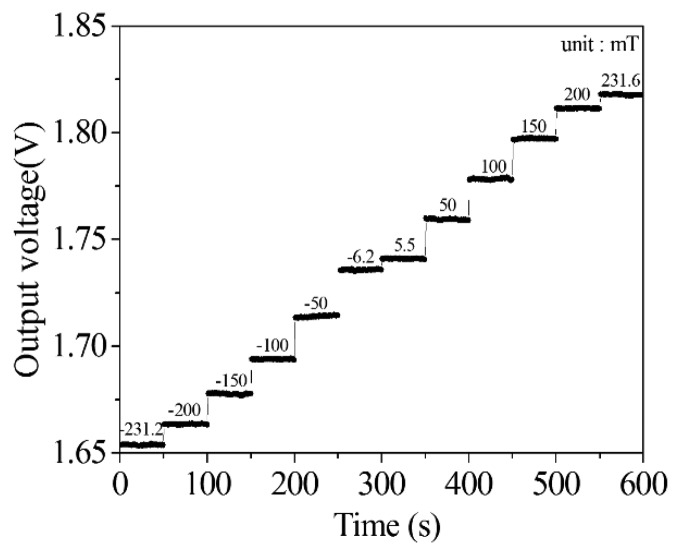
Output voltage of the magnetic sensor under different magnetic fields.

**Figure 13.**
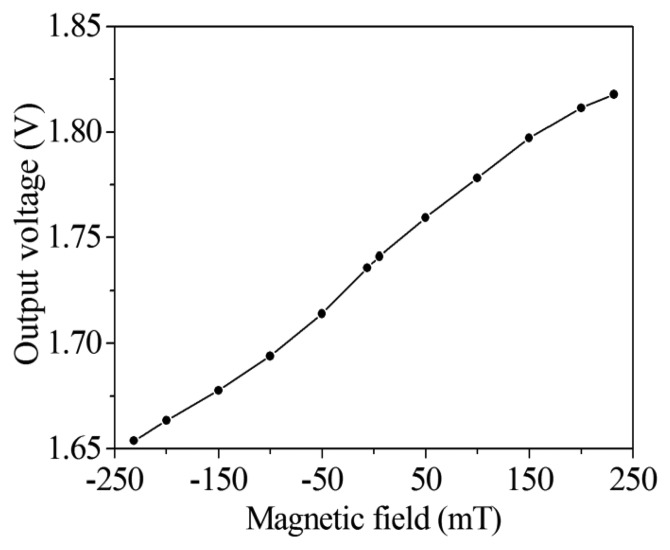
Relation between output voltage and magnetic field for the magnetic sensor.

The performances of the magnetic sensor with readout circuit were measured. A metal film was used to package the readout circuit for isolating the external magnetic field to the interference of the circuit performance. In order to reduce the energy dissipation, the operating condition of the magnetic sensor was set with a 2.6 V voltage supply and current supply of 4 mA, where the power consumption was 10.4 mW. The detecting range of magnetic field was set from −240 to 240 mT, because of working limitations of the magnetic field generator. Magnetic field intensity was changed by altering the current input of the magnetic field generator. Simultaneously, the magnetic field value was recorded by the Guess-meter, and the output voltage of the magnetic sensor was detected by the digital multi-meter. After recording data every 50 mT, the relation between the output voltage and magnetic field intensity were obtained. Figure 12 shows the output voltage of the magnetic sensor with readout circuit under different magnetic field intensities. The results showed that the output signal deviation of the magnetic sensor was quite small in the static magnetic field. Figure 13 depicts the relation between the output voltage and magnetic field for the magnetic sensor, and the results were obtained from the data in Figure 12. As shown in Figure 13, when the magnetic field varies from −240 to 240 mT, the output voltage of the magnetic sensor changes from 1.65 to 1.82 V. The sensitivity of the sensor is 354 mV/T. The evaluated results (Figure 7) are linear because we assume that the Hall mobility *μ_p_* in Equation (3) is constant in order to simplify the model. Actually, the Hall mobility depends on the external magnetic field. The Hall mobility generates a change when the external magnetic field varies [30]. Thereby, the measured results (Figure 13) are nonlinear. The nonlinearity is small, and the measured results approximate to the evaluated results.

Table 1 shows the properties of magnetic sensors from this work and the related research. Nazarinejad *et al.* [3] developed a GMI magnetic sensor with a special material of Co_73_Si_13_B_15_. This work does not need any special material, so the cost of the magnetic senor in this work is lower than that of Nazarinejad *et al.* [3]. Wattanasarn *et al.* [4] fabricated a 3D Lorentz force magnetic sensor using MEMS technology, and Estrada *et al.* [5] presented a MEMS-SOI 3D-magnetic field sensor. Their fabrications were complicated but the fabrication of the magnetic sensor in this work is easier than those of [4,5]. Ristic *et al.* [6] manufactured a magneto-transistor magnetic sensor using CMOS technology, and the sensor had a power consumption of 1,108 mW. The power consumption of the magnetic sensor in this work is lower than that of Ristic *et al.* [6]. Leepattarapongpan *et al.* [7] proposed a magneto-transistor magnetic sensor fabricated by CMOS technology, and the sensitivity of the sensor was 110 mV/T. The sensitivity of this work exceeds that of Leepattarapongpan *et al.* [7].

## 5. Conclusions

A magnetic microsensor has been designed based on a magneto-transistor and manufactured using the commercial 0.18 μm CMOS process. The fabrication of the magnetic sensor was compatible with the CMOS process, and without any post-process. The current of the magneto-transistor was transmitted from the emitter electrode to collector and base electrodes. The voltage difference of the bases in the magneto-transistor was small, therefore, a readout circuit was adopted to amplify the voltage difference of the bases and convert the signal into the output voltage. A voltage of 2.6 V and a current of 4 mA were supplied to the magnetic sensor, and the experiments showed that the output voltage of the magnetic sensor varied from 1.65 to 1.82 V as the magnetic field increased from −240 to 240 mT. The sensitivity of the magnetic sensor was 354 mV/T, and the power consumption was 10.4 mW.

## Figures and Tables

**Table 1.** Properties of magnetic sensors for this work and the related researches.

**Researchers**	**Sensing Principle**	**Sensitivity**	**Power Consumption**
Nazarinejad *et al.* [3]	GMI	20,000 Ω/T	–
Wattanasarn *et al.* [4]	Lorentz force	30.4 Ω/T	11 mW
Estrada *et al.* [5]	Hall	0.02 mV/T	–
Ristic *et al.* [6]	Magneto-transistor	80 μA/T	1,108 mW
Leepattarapongpan *et al.* [7]	Magneto-transistor	110 mV/T	–
This work	Magneto-transistor	354 mV/T	10.4 mW
